# Epicutaneous Application of Mannan Induces Psoriasis-like Inflammation in an Inbred Mouse Strain

**DOI:** 10.21769/BioProtoc.4845

**Published:** 2023-10-20

**Authors:** Huimei Wu, Kutty Selva Nandakumar

**Affiliations:** 1 Department of Pharmacy, the Eighth Affiliated City Hospital of Guangzhou Medical University, The Eighth People’s Hospital of Guangzhou, Guangzhou, China; 2 Southern Medical University – Karolinska Institute United Medical Inflammation Center, School of Pharmaceutical Sciences, Southern Medical University, Guangzhou, China; 3 Department of Environmental and Biosciences, School of Business, Innovation, and Sustainability, Halmstad University, Halmstad, Sweden

**Keywords:** Mannan, Psoriasis, Mouse model, Imiquimod, Inflammation

## Abstract

Mannan from yeast induces psoriasis-like inflammation in the skin of inbred mouse strains. Limitations of available models led us to develop a new psoriasis model with a rapid disease onset, severe disease course, short duration, and a simple and easy-to-induce protocol with much more practically convenient features and cost-benefits. Mannan-induced skin inflammation (MISI) is more severe than the classical imiquimod (IMQ)-induced skin inflammation (IISI), with characteristic features resembling human plaque psoriasis but with relatively fewer toxicity issues. Epicutaneous application of mannan (5 mg) in incomplete Freund’s adjuvant or Vaseline induces severe psoriasis in BALB/c female mice. Psoriasis area and severity index (PASI) and histological evaluation of the skin could help assess the disease development. MISI mimics natural environmental factors affecting the skin relatively more closely than IISI. This disease model can be used to dissect inflammatory pathways in the skin, identify genetic and environmental factors affecting psoriasis, and test potential pharmacological agents or new combinations of available drugs for treatment before designing clinical trials.

Key features

• *S. cerevisiae* mannan induces psoriasis-like skin inflammation (MISI) when applied on the skin of inbred mice.

• The MISI model has a rapid onset, severe disease, short duration, and simple and easy-to-induce protocol.

• MISI is more severe than imiquimod-induced skin inflammation (IISI).

• Female mice had a more severe disease than males in the MISI model, thereby allowing the study of sex-dependent disease mechanisms.

• The MISI model identifies skin inflammatory pathways and genetic/environmental factors affecting psoriasis.

• The MISI model can be used as a drug testing platform for potential pharmaceuticals to develop new therapeutics for psoriasis patients.

• The MISI model can be used to explore the relative contribution of different pattern recognition receptors in the development and severity of psoriasis.

**Graphical overview**


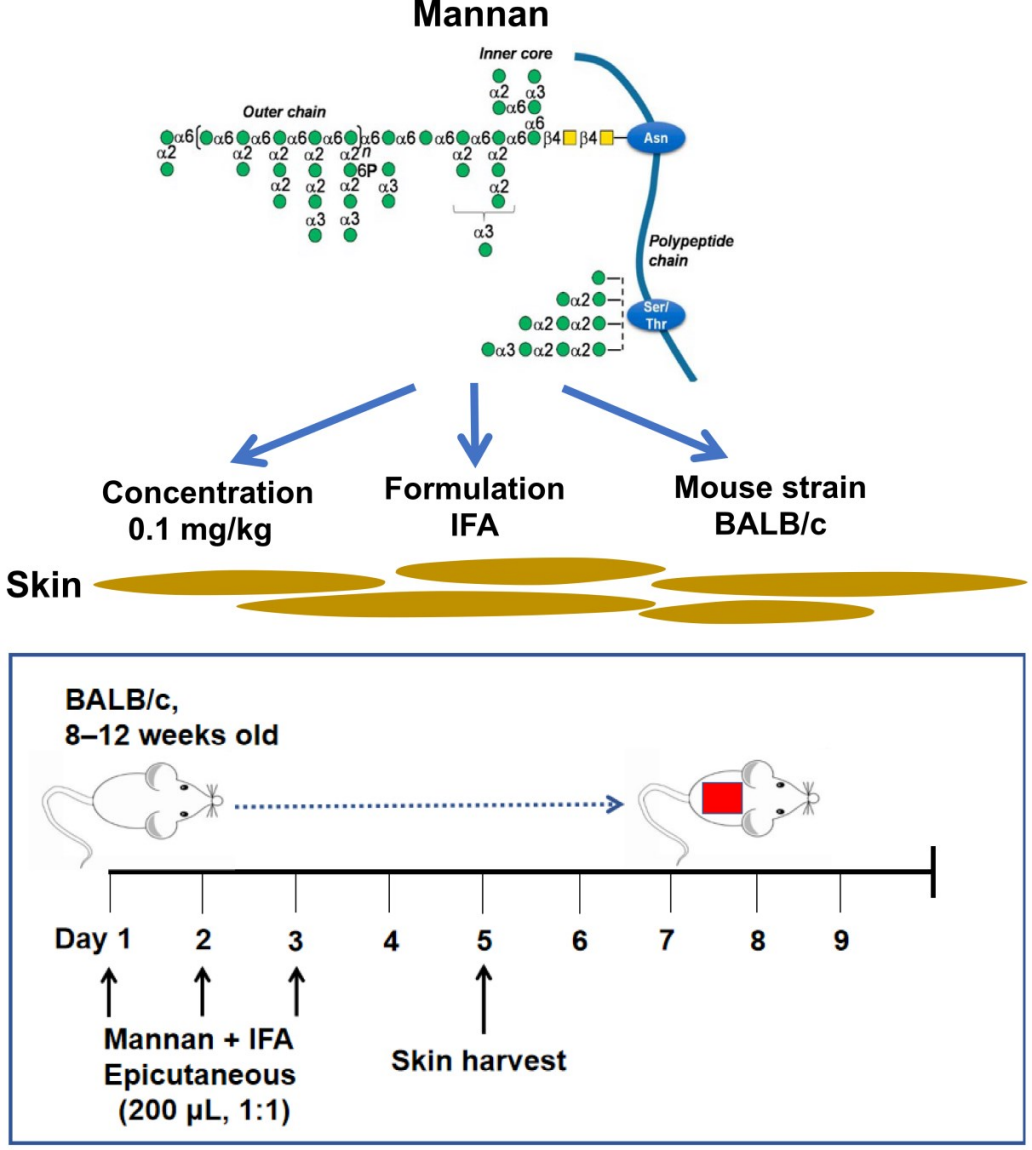


## Background

Psoriasis is a skin inflammatory disease affecting 3% of the population (Girolomoni et al., 2017; Zhong et al., 2018). Psoriasis etiology is still unclear, but a strong association with human leukocyte antigens (HLA) was documented (Knight et al., 2012; Tsoi et al., 2012). Environmental factors like sex, stress, and smoking affect the clinical manifestations of psoriasis (Fry and Baker, 2007). Keratinocyte hyperproliferation, increased blood vessel formation, and infiltration of immune cells are common in psoriasis skin (Van der Fits et al., 2009). Innate and adaptive immune responses are indispensable for disease development (Villanova et al., 2014). Proinflammatory cytokines and the immune cells secreting them activate the effector cells present in the skin. For example, T cell–derived cytokines like IL-17A and IL-17F activate keratinocytes, which produce anti-microbial peptides and more cytokines and chemokines. These inflammatory factors shape psoriasis skin inflammation (Ando et al., 2015).

**Figure 1.**
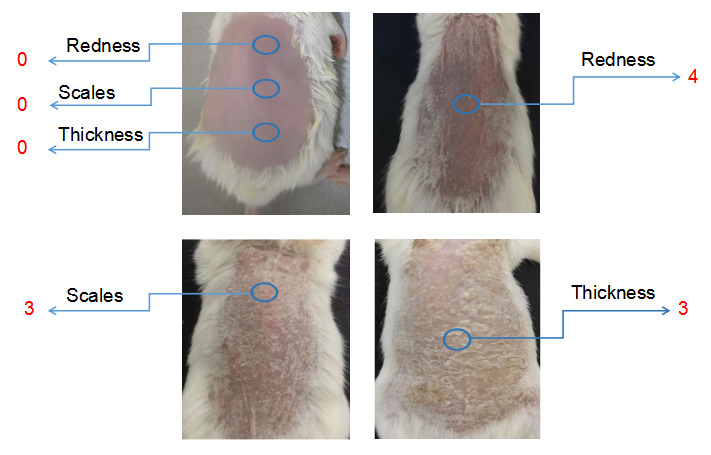
Clinical scoring for psoriasis inflammation. A representative psoriasis inflammation scoring method is given. PASI scores for the above skin conditions are as follows: redness: 4; scales: 3; thickness: 3. Each group contains 10 mice. BALB/c female mice were used for experiments. Left column pictures were reproduced from Figure 1A of the published paper (Wu et al., 2023).

Mannan is present in nearly all fungi species. *Saccharomyces cerevisiae* and *Candida albicans mannans* are pathogenic factors in psoriasis (Picciani et al., 2013). An intraperitoneal injection of mannan extracted from *Saccharomyces cerevisiae* cell wall was shown to induce psoriatic arthritis and psoriasis symptoms in reactive oxygen species (ROS)-deficient, *Ncf1*-gene-mutated mice that are available only in specific labs (Khmaladze et al., 2014). On the other hand, an epicutaneous application of imiquimod (IMQ) for 5–7 days leads to the development of an acute psoriasis phenotype. However, the disease developed in IMQ-induced psoriasis is often accompanied by severe weight loss in BALB/c mice (Swindell et al., 2017). Herein, we describe an induction protocol for plaque psoriasis-like inflammation in an inbred mouse strain (BALB/c) by epicutaneous application of mannan (Figure 1). A local (epicutaneous) instead of the intraperitoneal application of mannan could mimic a more natural way of skin exposure to environmental triggers for skin disease induction. Induced skin inflammation resembles human plaque psoriasis. Redness covered with white scales and skin thickness in the lesion area in mannan-exposed skin is more straightforward, making this model simpler for visually observing most manifestations of the disease.

Moreover, the proliferation of keratinocytes and innate immune cells, the infiltration of T lymphocytes in the skin, and an increased expression of proinflammatory cytokines were induced after epicutaneous mannan application (Wu et al., 2023). The activation of TLR7 and TLR8, mainly expressed in the immune cells, mediates the effects of IMQ (Bong et al., 2002). In contrast, mannan is a ligand for mannose receptors and is the primary trigger of IL-17 pathways in the host (Jiang et al., 2017). In addition, a higher-level expression of TLR2, TLR4, CD206, Dectin-2, Mincle, and DC-SIGN receptors was found in mannan-induced skin inflammation (MISI) (Wu et al., 2023). Thus, this disease model differs from IMQ-induced psoriasis-like inflammation, mainly in terms of the health status of the animals. Unlike the IMQ model, the body weight loss in MISI is negligible.

Furthermore, mannan had a shorter exposure time (3 days) to induce the disease than IMQ (5–7 days), demonstrating more cost-effective and convenient features. A response to dexamethasone in MISI has shown its usefulness in testing and validating various anti-psoriatic drugs for future treatments in psoriasis patients. Many autoimmune diseases show sexual dimorphism during disease development, and this plaque psoriasis-like skin inflammation induced by mannan also showed strong female preponderance, demonstrating its use in exploring sex-dependent mechanisms (Wu et al., 2022). Although mannan-induced skin inflammation results in acute and relapsing disease symptoms after repeated exposure, like other induced acute psoriasis mouse models, this model can also not completely mimic the relapsing and chronic characteristic features of human plaque psoriasis.

## Materials and reagents

**Biological materials**

1. 8–12-week-old BALB/c female mice maintained in a pathogen-free animal house were purchased from Southern Medical University and Guangdong Medical Animal Experiment Center. Each group contains 5–12 mice. All animal experiments were performed per the guidelines of the National Institutes of Health (NIH Publication No. 8023) and approved by the ethics committee of Southern Medical University (l2018183). Mice were kept in cages in a climate-controlled environment having 12:12 h light/dark cycles and given food and water ad libitum. Southern Medical University Animal Care and Use Committee, Guangzhou, China, approved all the procedures.
  BALB/c is among the most popular inbred mouse strains in many international research laboratories. The IMQ-induced psoriasis mouse model is commonly induced in these mice for drug evaluation. Skin redness can be more easily observed in this white mouse. BALB/c female mice were used in all the experiments described below.

**Reagents**

1. *S. cerevisiae* mannan (Sigma-Aldrich, catalog number: M7504) was dissolved (100 mg/mL solution) in sterile phosphate buffered saline, PBS (Gibco, catalog number: 14190-094).
2. Incomplete Freund’s adjuvant (IFA) (Sigma-Aldrich, catalog number: F5506-10)
3. Complete Freund’s adjuvant (CFA) (Sigma-Aldrich, catalog number: F5674)
4. Isoflurane (Healthcare, catalog number: 676544)
5. Phenobarbital (Sigma-Aldrich, catalog number: 57-30-7)
6. Imiquimod cream (Aldara^TM^, catalog number: 678432)
7. Hair removal cream (Nair, catalog number: 657789)
8. Paraffin (Leica, catalog number: 3960l095)
9. 4% Paraformaldehyde (Leagene, catalog number: DF0135)
10. Hematoxylin solution (Mayer) (Beyotime, catalog number: C0105)
11. 0.2% Eosin solution in PBS (Mayer) (Beyotime, catalog number: C0105)
12. Xylene solution (Beyotime, catalog number: C0105)
13. Neutral gum (Solarbio, catalog number: G8590)
14. Vaseline (Yuanye, catalog number: 8009-03-8)
15. Liquid Paraffin oil (Yuanye, catalog number: 67552)
16. Ethanol (absolute) (Mackline, catalog number: E821482)
17. Plus-loaded slides (Thermo Fisher, catalog number: 6885)
18. Xylene-based mounting medium (HistoLab, catalog number: 00801)

**Solutions**

1. Different concentrations of ethanol (see Recipes)
2. Mannan-PBS solution (see Recipes)
3. Phenobarbital-normal saline solution (see Recipes)
4. Preparation of mixture (see Recipes)

**Recipes**

1. **Different concentrations of ethanol**
  **Table:** 

Reagent	Ethanol (absolute)	H_2_O
95% ethanol	950 mL	50 mL
90% ethanol	900 mL	100 mL
80% ethanol	800 mL	200 mL
70% ethanol	700 mL	300 mL
50% ethanol	500 mL	500 mL
2. **Mannan-PBS solution**
  **Table:** 

Solution	100 mg/mL mannan-PBS solution	PBS
50 mg/mL mannan-PBS solution	50 μL	50 μL
3. **Phenobarbital-normal saline solution**
  **Table:** 

Solution	Phenobarbital powder	Normal saline
80 mg/mL phenobarbital-saline solution (5 mL)	400 mg	5 mL
4. **Preparation of mixture**
  **Table:** 

Mixture	Mannan-PBS solution (50 mg/mL)	IFA
Mannan + IFA	100 μL	100 μL

## Equipment

1. Small animal weighing device (CHIKO, model: ZK-DST)
2. Digital vernier caliper (Yasuwang, catalog number: ASO-1-894-01)
3. Mouse skin shaver (Riward, catalog number: CP-5200)
4. Tissue processing/embedding cassettes with lid (Sigma-Aldrich, catalog number: Z672122)
5. Brush (Asone, catalog number: CC-5667-04)
6. Vacuum tissue processor (Leica, model: ASP300S)
7. Embedding machine (Tissue-Tek; Sakura)
8. Microtome (Leica, model: RM2255)
9. Eclipse upright optical microscope (Nikon, model: Ci-E)

## Software and datasets

1. Image-pro plus 6.0 software (Nikon; Ci-E)
2. GraphPad Prism 5 (GraphPad Prism; version 5)

## Procedure

**Basic Protocol 1**

**Mannan-induced skin inflammation**

Epicutaneous application of 50 μL of mannan mixture with incomplete Freund’s adjuvant at the back of the BALB/c female mice for three days induces psoriasis-like skin inflammation. Monitoring skin redness, scales, and thickness daily for 7–9 days or until the inflammation subsides using the scoring protocol described in Support Protocol 1 is essential. Before starting any animal experiment, apply for and get valid ethical permits from ethical review boards of respective institutions/regions.

**Protocol steps—step annotations**

- **Preparation of mannan stock solution**
  1. Dissolve the mannan powder (5 g/vial) by gently adding 2 mL of sterile PBS and then transfer all the contents into a 50 mL centrifuge tube, adding another 48 mL of PBS to get a 100 mg/mL concentration.
*Note: Avoid foam formation, though occasional shaking of the solution is required to dissolve the powder completely.*
  2. Aliquot and label the mannan solution in sterile 10 mL tubes and freeze them at -20 °C until used.
*Note: Mannan solution at 100 mg/mL concentration is stable for over 12 months if stored at -20 °C.*
- **Epicutaneous application of mannan for psoriasis induction**
  1. To get consistent results, 8–12-week-old BALB/c female mice (5–10 mice/group) from specific pathogen-free breeding centers are required. Acclimatize the mice for at least one week before starting the experiment.
  2. Use at least five mice per cage in a climate-controlled environment with 12:12 h light/dark cycles; provide food and water ad libitum.
  3. Use mice epicutaneously treated with PBS on the back, similarly to the mannan application (minimum five mice), for control.
  4. Thaw 1 mL of mannan-PBS solution, previously stored in the freezer, at room temperature for 15 min.
*Note: Avoid frequent freeze-thaw cycles. The volume of mannan thawed depends on the number of mice used in an experiment.*
  5. Remove the hair from the mouse skin at the back (2.0 cm × 3.5 cm area) first by using a small mouse shaver, which has a 2.4 cm wide blade that can cut as close as 0.1 mm to the skin.
*Note: Avoid making wounds on the skin surface.*
  6. Use the hair removal cream to remove the remaining hair from the specified area of the mouse skin. Take the hair removal cream on a smooth plastic plate and then smear the cream on the surface of the skin. Wait for 20 min and finally remove the cream from the hair using a paper towel.
  7. Accurately weigh the mice and give an intraperitoneal injection of 80 mg/mL phenobarbital-normal saline solution to anesthetize mice.
*Note: The weight of mice is measured in grams and accurate to one decimal place, and intraperitoneal injection of phenobarbital-normal saline solution should be no more than 200 μL in volume.*
  8. Mix 100 μL of mannan-PBS solution (50 mg/mL) with IFA (100 μL) and apply mannan + IFA mixture for each mouse. Place mannan-PBS solution at the bottom of a 1.5 mL centrifuge tube, and then add IFA gently; use a pipette to mix mannan-PBS and IFA until the mixture becomes an emulsion. Treat at least five mice with the prepared mixture and use another five mice treated with PBS as a control.
*Note: Mannan and IFA mixture need to be prepared before each experiment, and homogeneous mixture preparation is required before application on the back of mice to get reproducible results.*
  9. Smear the mixture on the back of the mouse uniformly using a cotton swab pre-soaked in PBS.
*Note: Apply a thin layer of the mixture on the skin and avoid applying the mixture too much to any one place. Use a fine brush (natural or synthetic, but not foam) for application. Applying a higher concentration (10 mg) of mannan leads to acute inflammatory reactions, and the scales will peel off quickly, hence not optimal for psoriasis induction.*
  10. Repeat steps B8 and B9 for three consecutive days.
*Note: Apply mannan-IFA mixture at 9:00–10:00 am for three days. Remember to apply mixture at 24-hour intervals.*
  11. Score the psoriasis lesions daily by following Support Protocol 1.
*Note: The disease progression and scoring start 48 h after mannan treatment for the first time and should be monitored every 24 h for 9 days. Observe redness, scales, and thickness from day 2 onwards. Redness and scales are typically apparent after days 3 and 4, respectively. These psoriasis symptoms reach maximum severity on day 5, and the mice gradually start recovering from day 7 onwards. In most inbred mouse strains (BALB/c, C57Bl/6J, C57Bl/6NQ, KM, DBA/1, ICR, and NIH), signs of inflammation could be observed in naïve mice exposed to mannan under experimental conditions described herein (Wu et al., 2022 and 2023).*
**Support Protocol 1**
**Psoriasis Area and Severity Index (PASI) scoring protocol**
  After mannan application, mice should be inspected daily for disease symptoms, as shown in Figures 1–5. PASI involves scores for redness (0–4), scales (0–4), and thickness (0–4). The standard of PASI scoring was reported earlier (Van der Fits et al., 2009). The total score of PASI (maximum disease severity) per mouse is 12, as shown in the psoriasis inflammation scoring protocol (Table 1). Score redness and scales visually and measure skin thickness using a digital vernier caliper.
  **Table 1.** Scoring standards of PASI for psoriasis inflammation in mice

Scores of psoriasis	0	1	2	3	4
Redness	No	Mild	Moderate	Severe	Very severe
Scales	No	Mild	Moderate	Severe	Very severe
Thickness	No	Mild	Moderate	Severe	Very severe
**Protocol steps—step annotations**
  1. Gently take each mouse from its cage and observe the back of the mice for psoriasis progression.
*Note: The critical components of the PASI scoring are outlined in Table 1.*
  2. Evaluate the redness using a 0–4 scale for mannan and PBS-applied mice during disease. Example images are given below for redness scores (Figure 2).
    **Figure 2.**
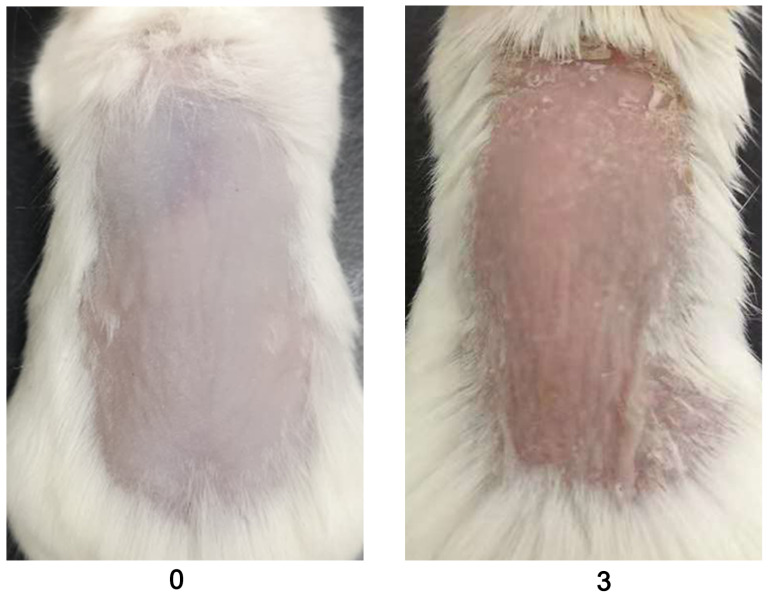
Scores for redness (0 and 3) are given by observing the back skin of the mice. An example is shown in the above figure (n = 10/group). BALB/c female mice were used for experiments. Right column images with 3 points were reproduced from Figure 1A of the published paper (Wu et al., 2022).
  3. Evaluate the scales using a 0–4 scale for mannan and PBS-applied mice during disease. Example images are given below for scoring the scales (Figure 3).
    **Figure 3.**
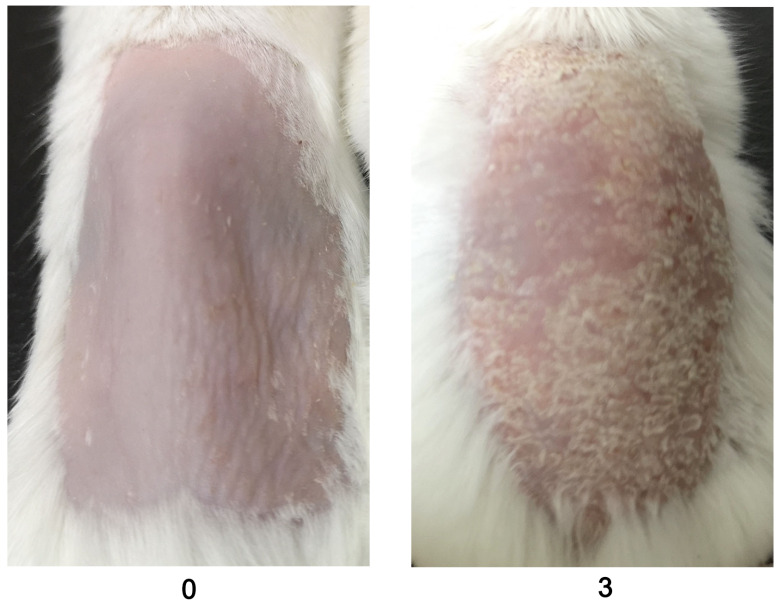
An example was given for scale scores (0 and 3) by observing the back skin of the BALB/c female mice in mannan-induced skin inflammation (MISI) (n = 10/group). The right column picture was reproduced from Figure 1A of the published paper (Wu et al., 2022).
  4. Measure the skin thickness with a caliper daily.
  5. Choose the same place and angle on the skin for measuring thickness, using a digital vernier caliper (0.1 mm = 1 score), above that of naïve mouse skin, scored as 0 (Figure 4).
    **Figure 4.**
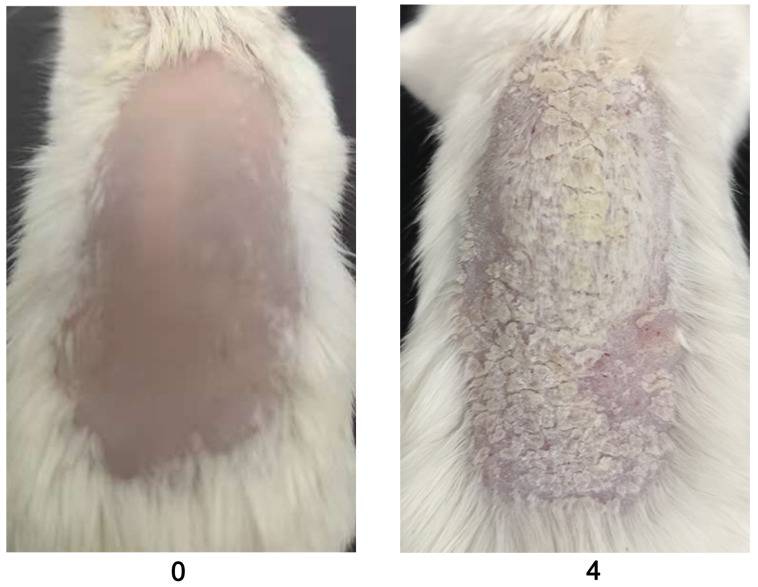
Representative example for skin thickness scores (0 and 4) in BALB/c female mice after PBS or mannan treatment (n = 10/group). Skin thickness is measured using a vernier caliper.
  6. Perform scoring procedures daily for psoriasis development (refer to Figure 5 and Table 2).
*Note: The incidence of psoriasis is generally very high (100%) and consistent from experiment to experiment. However, disease severity is strain dependent. The most disease-prone strains are BALB/c and C57Bl/6 mice.*
  **Figure 5.**
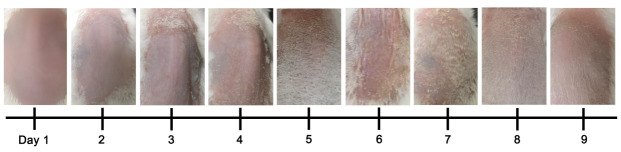
Development of psoriasis in mannan-induced psoriasis from days 1 to 9 in BALB/c female mice (n = 10 mice/group). The image of day 6 was reproduced from Figure 1A of the published paper (Wu et al., 2022).
  **Table 2.** An example of individual and total PASI scores in BALB/c female mice from days 1 to 9 in MISI

Day	Redness	Scales	Thickness	PASI
1	0.5	0	0	0.5
2	1.2	1.0	0.8	3
3	2.0	2.0	1.9	5.9
4	2.5	2.8	2.5	7.8
5	3.5	4	3.5	11.0
6	2.5	2.8	2.8	7.1
7	1.5	1.8	1.9	5.2
8	1.0	1.5	1.0	3.5
9	1.0	0.5	0.5	2.0
  Skin starts to show redness from day 1 and scales from day 2 after the mannan-IFA mixture application, with a maximum psoriasis severity at days 4 or 5. Symptoms of psoriasis disappear entirely around day 9. A second cycle of exposing the mice to mannan on days 10–12 induced even more severe psoriasis symptoms than the first cycle of exposure (Wu et al., 2023).

**Basic Protocol 2**

**Imiquimod-induced skin inflammation (IISI)**

Imiquimod (IMQ) treats keratosis, basal cell carcinoma, and external genital/anal warts. However, topical treatment with IMQ induced typical psoriasis-like inflammation, including increased epidermal thickness, erythema, and skin thickness (Cai et al., 2011). IMQ is a toll-like receptor-7 and -8 (TLR7 and 8) ligand and can exacerbate psoriasis development in patients, possibly acting via the IL-17/IL-23 axis.

**Protocol steps—step annotations**

1. Divide IMQ cream from a 250 mg pack into five portions.
*Note: Preparing equal portions of Aldara cream may sometimes be challenging to achieve. Hence, careful attention is required to have a constant amount of IMQ smeared on the back of mice.*
2. Thirty-six mice were randomly divided into three groups containing PBS, mannan, and IMQ groups. Remove the fur using a mouse shaver and hair removal cream from a 2 cm × 3.5 cm area of the back of the mouse.
*Note: Avoid making wounds on the skin surface.*
3. Accurately weigh the mice and give an intraperitoneal injection of 80 mg/mL phenobarbital-natural saline solution to anesthetize mice.
*Note: The weight of mice is measured in grams and accurate to one decimal place, and intraperitoneal injection of phenobarbital-natural saline solution should be no more than 200 μL in volume.*
4. Apply 50 mg of IMQ cream on the back of the mouse from 9:00 to 10:00 am daily for five days consecutively.
*Note: Apply the cream at 24 h intervals for five days. Apply a thin coat of IMQ cream on the skin and avoid applying too much cream to any one place. Use a fine brush (natural or synthetic, but not foam) to apply the IMQ cream.*
5. Score mice’s redness, scales, and thickness before applying IMQ using Support Protocol 1.
*Notes:*
  - *A similar disease course was observed in MISI and IISI, as shown in Figure 6. Individual scores and total PASI in MISI (day 5) and IISI (day 5) were given in Table 3.*
  - *There is negligible weight loss after mannan application, while in the IMQ-induced psoriasis model, 12%–15% loss of initial weight was observed (Wu et al., 2022).*
    **Figure 6.**
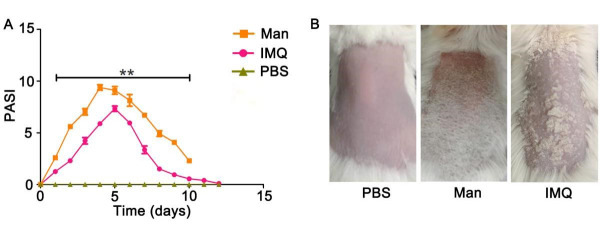
Comparison of psoriasis area and severity index (PASI) scores and clinical disease of mannan with imiquimod (IMQ)-induced psoriasis. PASI and psoriasis disease in female BALB/c mice. (A) PASI scores of PBS- mannan and IMQ-induced psoriasis-like skin inflammation (n = 12/group). (B) Representative images of PBS-, mannan-, and IMQ-applied mice at the peak of psoriasis (day 5) were shown. The data represent mean ± SEM. **, p < 0.01.
    **Table 3.** Individual and total PASI scores in BALB/c female mice after mannan or IMQ application

Scores	PBS	Mannan	IMQ
Redness	0.5	2.5	3.2
Scales	0	3	2.5
Thickness	0	3	2.5
PASI	0.5	8.5	8.2

**Basic Protocol 3**

**Histological staining of psoriasis skin**

Use hematoxylin and eosin staining to assess the skin morphology from psoriasis and naïve mice. Hematoxylin has a negative charge, and it is alkaline, which gives it a blue color after binding with the nucleus. Eosin binds with protein cations so that the cytoplasm is stained red. Hematoxylin and Eosin (H&E) staining can show the epidermis’ hyper-proliferation and immune cell infiltration in the epidermis and dermis. Determine the epidermal thickness by measuring the average interfollicular distance under the microscope in a blinded manner. Histopathological evaluation of diseased skin sections shows hyperkeratosis and acanthosis, elongated “rete-like” ridges, and infiltration of immune cells in the diseased skin.

**Protocol steps—step annotations**

1. Cut a specified area (2 cm × 3.5 cm) of the mouse skin and prepare for the histology.
2. Fix the skin by submerging it in 4% paraformaldehyde for 24 h at 4 °C. Then, cut the skin into 1 cm × 1 cm pieces using scissors and tweezers.
*Note: Adequate fixing of the tissues is essential to avoid stiff and brittle specimens because of dehydration and tissue processing steps. Several factors (quality of buffer, penetration into tissues, volume, temperature, concentration, and time) can affect this fixation step.*
3. Dehydrate the skin tissues in different concentrations of alcohol for 12 h using Support Protocol 2.
4. Embed the dehydrated skin in paraffin using 1 cm × 1 cm skin pieces in a plastic paraffin-embedding cassette.
5. Cut the tissue into 8 mm sections using a tissue microtome at room temperature.
*Note: Use longitudinal instead of cross-sectional paraffin sections and then store them at -20 °C before staining.*
6. Stain the skin sections with H&E by using Support Protocol 3.
*Note: Use plus-loaded slides (for example, Fisherbrand^TM^ Superfrost^TM^ Plus microscope slides) for electrostatic adherence of tissue sections to the glass without adhesives or protein coatings. This step will avoid the loss of tissue sections from the slides during the staining process.*

**Support Protocol 2**

Dehydrate the skin tissues through graded ethanol baths. Next, skin samples are made transparent in xylene solution, immersed in 75 °C paraffin oil separately, and embedded in the paraffin-embedding cassettes, which are stable for many years. Paraffin wax is a mixture of n-alkanes with a carbon chain length between 20 and 40 that is solid at room temperature but melts at temperatures between 65 °C and 70 °C.

**Protocol steps—step annotations**

Dehydrate the skin samples as follows:

1. Dehydrate in 50% ethanol for 60 min.
2. Dehydrate in 70% ethanol for 120 min.
3. Dehydrate in 80% ethanol for 120 min.
4. Dehydrate in 90% ethanol for 90 min.
5. Dehydrate in 95% ethanol for 90 min.
6. Dehydrate in 95% ethanol for 30 min.
7. Dehydrate in 100% ethanol for 40 min.
8. Dehydrate in 100% ethanol for 30 min.
9. Rehydrate the skin sections in xylene solution with two changes for 10 min each.
10. Immerse the skin sections in 75 °C paraffin oil with two changes for two hours each.

**Support Protocol 3**

**H&E staining of skin samples**

The H&E stain is one of the primary medical diagnostic stains performed with formalin-fixed skin samples. This stain gives a candid picture of the different cells and structures in the skin. Hematoxylin (a complex of aluminum ions and oxidized hematoxylin) stains nuclei and keratohyalin granules in blue. The nuclear membrane will stain dark blue. The counterstain, eosin, gives a pink/orange color. Eosin stains red blood cells as a dark shade, muscles, and connective tissues as light orange, and the cytoplasm as a faint pink. Hematoxylin and eosin may be diluted in water and ethanol, respectively, as required.

**Protocol steps—step annotations**

Submerge the skin samples in each solution as described below:

1. Deparaffinize the skin sections in xylene solution with two changes for 10 min each.
2. Rehydrate them in absolute ethanol for 5 min.
3. Rehydrate them in 95% ethanol for 5 min.
4. Rehydrate them in 85% ethanol for 5 min.
5. Rehydrate them in 75% ethanol for 5 min.
6. Wash the slides in flowing water for 3 min.
7. Stain the slides with Mayer’s hematoxylin solution for 8 min.
8. Wash the slides in running tap water for 5 min.
*Note: Do not stain the sections with excessive hematoxylin; if it happens, then wash the slides with 1% H_2_O_2_ in 70% ethanol.*
9. Counterstain the slides using 0.2% eosin in PBS for 2 min.
10. Dehydrate the slides in 75% ethanol for 5 min.
11. Dehydrate the slides in absolute ethanol for 5 min.
12. Treat the slides with xylene solution, two changes for 5 min each.
13. Mount the slides with a xylene-based mounting medium.

**Figure 7.**
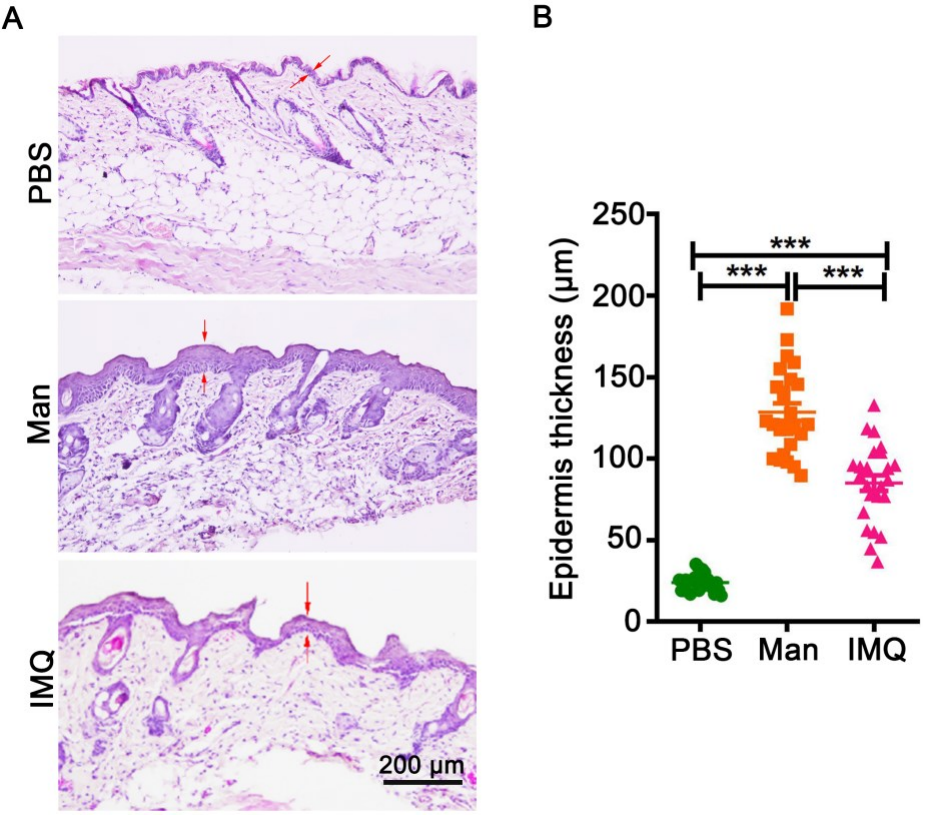
Histological staining of skin and measurement of epidermal thickness. (A) Representative pictures of histology staining of skin from mice applied with PBS, mannan, or imiquimod (IMQ) (n = 5/group). (B) Measured five different places of the epidermis to calculate mean thickness values. BALB/c female mice were used for experiments. Red arrows were used for marked epidermis thickness. Scale bar = 200 μm. The data represent mean ± SEM. ***, p < 0.001.

*Note: Xylene is toxic (Kandyala et al., 2010), and commercially available alternatives can be used (Falkeholm et al., 2001). Representative examples of H&E-stained skin sections are shown in Figure 7a–7c.*

**Support Protocol 4**

**Measurement of epidermal thickness**

**Figure 8.**
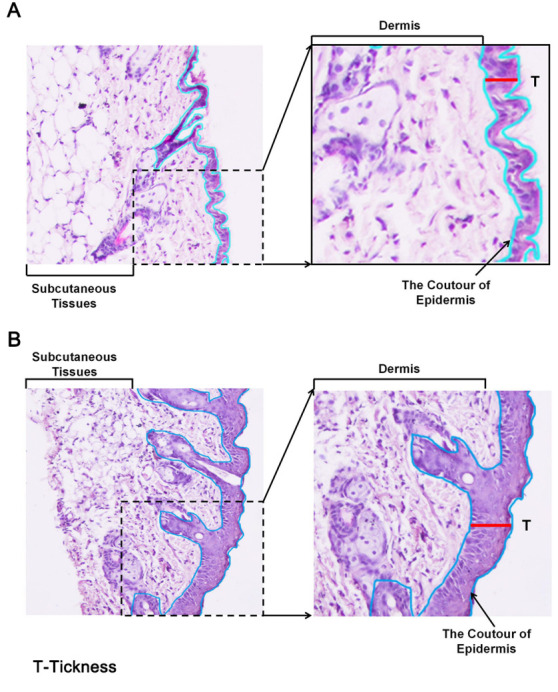
Measurement of epidermal thickness in mice using a microscope and image analysis software. Example for measuring epidermis thickness of skin in histological images in (A) PBS- or (B) mannan-exposed mice. The red line was used for marking and measuring epidermis thickness. Left column pictures were captured at 20× magnification using an eclipse upright optical microscope. BALB/c female mice were used for experiments. Skin samples were harvested on day 4 from PBS- and mannan-treated groups.

Measure the epidermal thickness by light microscopy, which is a reliable and reproducible method. However, sample collection methods and different sample processing procedures for histology, like fixation, dehydration, embedding, and staining, may alter the tissues, leading to inaccurate thickness measurements. Hence, control and diseased skin samples collected and processed in the same way should be measured simultaneously to minimize such issues. Measure the thickness of the epidermis with a computerized light microscope system (Figure 8), which performs systematic area measurements in sections. The images thus obtained can be analyzed using an image analysis software like Image-pro plus.

**Protocol steps—step annotations**

1. Measure the thickness of the epidermis under an eclipse upright optical microscope using Image-pro plus (Nikon, Japan) in at least five different tissue areas to measure the thickness accurately. Show mean thickness values from an experiment as shown in Figure 7B.
2. For measuring epidermal thickness, open Image-pro plus 6.0 software and select the line tool.
3. Draw a line of unit length on the ruler.
4. Select *Analyze* → *Set Scale*. Enter the length of the line drawn in the known distance, the unit of the length in input unit, check the Global checkbox (use this standard format for all pictures) and click OK.
5. Select the length to measure based on the line in the picture. Choose at least three images from three different mice for mannan-induced psoriasis skin. Choose 10 places to estimate the size of the epidermis. Use similar protocols for PBS and IMQ groups as well. Obtain at least 30 measurements for the epidermis from each group. Analyze the difference between groups using an unpaired *t*-test.
6. Press Ctrl + M (Measurement) to display the results in the results window.
7. Use GraphPad Prism, version 5, to analyze the data.
8. Use at least five sections from one mouse, and five mice per group, for measurements.
9. Measure the thickness of skin sections from psoriasis and naïve mice skin sections in parallel.
10. Blindly take the measurements to avoid experimenter bias, and the thickness values can be expressed as mean ± SEM.

## Data analysis

The data were analyzed with GraphPad Prism 5 and are presented as mean ± SEM. Two-tailed unpaired Student’s *t*-test was used for comparing PASI between mannan- and IMQ-exposed mice. One-way analysis of variance (ANOVA) with Bonferroni or Newman–Keuls correction was used to compare epidermis thickness between PBS, mannan, and IMQ groups. Probability values < 0.05 were considered significant at a 95% confidence interval.

## Validation of protocol

The severity of mannan-induced skin inflammation was much higher than imiquimod-induced psoriasis-like skin inflammation (Wu et al., 2023), and female mice developed more severe psoriasis symptoms than males (Wu et al., 2022). Neutrophil cytosol factor (*Ncf1*), which encodes p47phox, is critically involved in NADPH oxidase 2 (NOX2) complex formation. The NOX2 complex is responsible for the induction of reactive oxygen species (ROS) and is an essential regulator of several autoimmune diseases. Epicutaneous exposure to mannan induced a severe, aggravated disease in mice having either spontaneous Ncf1^m1J^ mutation or Ncf^90H^ mice having a major human single nucleotide variant on the *Ncf1* gene, causing an amino acid replacement from arginine to histidine at position 90 (Li et al., 2023).

## General notes and troubleshooting

**General notes**

- The usage of a digital vernier caliper for measuring mouse skin thickness of psoriasis skin:
  Gently pinch a small piece of skin with your left hand, hold the electronic vernier caliper with your right hand, and press the skin vertically downwards into the outer measuring claw. Gently slide the movable end of the outer measuring claw to make it tightly grip the skin and avoid damaging it. Ensure that each mouse’s skin thickness is measured and calculated similarly. The skin thickness of the PBS group can be used as a negative control.
- The usage of isoflurane in anesthetizing experimental mice:
  Place three cotton balls into a 50 mL centrifuge tube and soak the cotton balls with isoflurane. Place the mouse head into the centrifuge tube for 30 s when anesthetizing. Moreover, note that the time should not exceed 60 s. Alternatively, a commercially available isoflurane anesthetizing machine can be used.
*Note: Phenobarbital, a barbituric acid derivative, is a long-acting sedative and an anticonvulsant (Suddock et al., 2023). It acts as a non-selective central nervous system depressant. It promotes binding to inhibitory gamma-aminobutyric acid (GABA) subtype receptors and modulates chloride currents through receptor channels. It also inhibits glutamate-induced depolarizations. Whereas isoflurane is an inhaled, short-acting general anesthetic used in surgery (Miller et al., 2023). It is used for induction and maintenance of general anesthesia. It induces muscle relaxation and reduces pain sensitivity by altering tissue excitability. Isoflurane decreases the extent of gap junction-mediated cell-cell coupling and alters the activity of the channels that underlie the action potential. It also activates calcium-dependent ATPase in the sarcoplasmic reticulum by increasing the fluidity of the lipid membrane. Isoflurane binds to the GABA receptor, the large conductance Ca^2+^-activated potassium channel, and the glutamate and glycine receptors.*
- Skin paraffin-embedding method for H&E staining.
  1.0 cm × 1.0 cm skin is vertically embedded in a paraffin-embedding cassette after dehydration. Pour liquid paraffin at 70 °C into the cassette and place it on ice for cooling. Note that the skin must be placed vertically in the embedding cassette.

**Troubleshooting (Table 4)**

**Table 4.** Troubleshooting

Possible questions	Solutions
Psoriasis does not develop after mannan application.	• Make sure that 100 mg/mL mannan-PBS solutions are stored in a -20 °C freezer. • Deeply anesthetize mice with phenobarbital for 40 min before mannan-IFA mixture applications. • Make sure that all mice for experiments are at least 8 weeks old.
Mannan-PBS solution and IFA are unable to mix evenly.	• Take mannan solution into 1.5 mL tubes at first, and then add IFA gently. Use 1 mL syringes to draw mannan solution and mix it with IFA for at least 20 times.
Mice dead after IMQ application.	• Make sure that all mice for experiments are at least 8 weeks old. • Weigh the IMQ cream accurately and no more than 62.5 mg/mice per day should be applied.
Severe weight loss of mice after IMQ application.	• Replenish water and food at regular intervals, and provide edible nuts, if necessary, on bedding material in the cages to avoid excess stress to the animals.
Wounds in the skin due to shaving before the experiments.	• Use a small, serrated shaver available commercially.
Fighting between animals during experimental period.	• The number of mice kept per cage must follow ethical principles and should not exceed the maximum allowed based on cage space available per mouse. • Generally, do not mix mice from different breeding cages after weaning them. So, the number of mice per cage must be calculated and housed before the weaning stage. • Do not place male mice in cages used for female mice, even while changing the mice to new cages during experiments or the scoring phase. Male mice get stressed if exposed to materials used for placing female mice and will start fighting, creating wounds in the skin and tail areas. • Fighting between female mice might be due to puberty-related issues and maneuvering for dominance. Since mice can live in groups a pecking order is normally established. For the most part female mice usually live peacefully. Lack of enrichment, such as enough places to hide, exercise wheels, and toys can be aggravating factors. • If female mice are fighting, transfer them to a new cage together because the aggression is, in general, based on the familiar smells the mice have that remind them of their territory and where they fit within the group.
